# Hickam’s Dictum Prevails: Kaposi Sarcoma and Primary Effusion Lymphoma in a Person Living With Human Immunodeficiency Virus (HIV) and Human Herpesvirus 8 (HHV-8) Infection

**DOI:** 10.7759/cureus.78081

**Published:** 2025-01-27

**Authors:** Arthur E McKinnon, Sahil Maharaj, Muhammad I Randeree, Sithembiso A Sikhosana

**Affiliations:** 1 Internal Medicine, Rob Ferreira Tertiary Hospital, Nelspruit, ZAF; 2 Radiology, Rob Ferreira Tertiary Hospital, Nelspruit, ZAF

**Keywords:** advanced hiv disease, disseminated kaposi sarcoma, epstein- barr virus, human herpes virus type 8, human immunodeficiency virus infection, kaposi's sarcoma-associated herpesvirus (kshv), primary effusion lymphoma, r-chop therapy, south africa, sub-saharan arica

## Abstract

Human immunodeficiency virus (HIV) infection has a plethora of effects on its host, including increased susceptibility to opportunistic infections and increased likelihood of developing HIV-associated malignancies. The combined cellular dysfunction orchestrated by co-infection with oncogenic viruses, such as human herpesvirus-8 (HHV-8) and Epstein-Barr virus (EBV), further amplifies the risk of malignancy in people living with HIV/AIDS (PLHA). We report a rare case of Kaposi sarcoma (KS) and extra cavitary primary effusion lymphoma (EPEL) in a 48-year-old woman with advanced HIV disease on antiretroviral treatment presenting with arthralgia, generalized body weakness, drenching night sweats, cavitary effusions, a violaceous rash on her left leg, as well as generalized lymphadenopathy. This report highlights the shared etiological role of HHV-8 in PLHA with both KS and EPEL. Given the rarity of the dual presentation of these conditions, it shows that the prudent application of Occam’s razor may suffice in the majority of cases. However, Hickam’s dictum should be applied when such diagnostic dilemmas exist.

## Introduction

The coexistence of Kaposi sarcoma (KS) and primary effusion lymphoma (PEL) is a rare phenomenon, mostly reported in case reports. Currently, there exists no real epidemiological data on this rare presentation.

Human immunodeficiency virus (HIV) is known to significantly increase the risk of developing malignancies due to profound immunosuppression [1]. People living with HIV/AIDS (PLHA) are 500 times more likely to be diagnosed with KS and 12 times more likely to be diagnosed with non-Hodgkin lymphoma (NHL), when compared to the general population [2]. The advent of antiretroviral therapy (ART) has markedly reduced the incidence of opportunistic infections. However, HIV-associated malignancies, particularly AIDS-defining cancers such as KS and PEL, remain significant causes of morbidity and mortality [3,4]. 

The risk of cancer in PLHA is driven by persistent immunosuppression, chronic antigen stimulation, and inflammation. Co-infection with oncogenic viruses, such as human herpesvirus-8 (HHV-8) and Epstein-Barr virus (EBV), also increases the likelihood of developing malignancy [5]. The combined orchestration of these factors is central to understanding the pathogenesis of both KS and PEL. 

KS is a vascular tumor strongly associated with HHV-8, also known as Kaposi sarcoma-associated herpesvirus (KSHV). It typically presents with violaceous, nodular, or macular skin lesions but may also involve mucosal surfaces, the gastrointestinal tract, and pulmonary systems [6].

PEL is a rare and aggressive B-cell lymphoma that characteristically presents as malignant effusions in serous body cavities (pleural, peritoneal, or pericardial) without a detectable solid tumor mass. Less frequently, PEL can present with extra cavitary disease, such as lymphoma in the lymph nodes and extranodal sites, referred to as extra cavitary PEL (EPEL) [7]. PEL accounts for approximately 4% of HIV-associated NHL. Symptoms are often related to the effusions, such as dyspnoea, abdominal distension, or chest pain. Constitutional B symptoms, such as fever, weight loss, and night sweats are common [7,8].

The coexistence of KS and PEL in PLHA highlights the shared etiological role of HHV-8. This dual pathology underscores the profound immunosuppressive state in advanced HIV disease (AHD) and the synergistic oncogenic potential of HIV, HHV-8, and EBV. These malignancies may manifest concurrently or sequentially, with overlapping clinical and pathological features. We report a rare case of KS and EPEL presenting concurrently in a woman with AHD. 

## Case presentation

A 48-year-old woman with AHD and a CD4 count of 167 cells/µL, WHO stage IV, on ART presented to our hospital which provides a tertiary service to neighboring medical facilities in a rural province in South Africa. The patient's ART regimen included tenofovir 300 mg, lamivudine 300 mg, and dolutegravir 50 mg as a daily fixed-dose combination. She had taken ART for four years prior to this presentation. The patient’s main complaints included a two-month history of arthralgia, generalized body weakness, drenching night sweats, swelling of her abdomen and feet, a purple rash on her left leg, as well as lumps growing in her neck, axillae, and groin region. The rash started as one purple spot on her left lower leg about two months ago and continued to spread, affecting the majority of the skin of her left leg. She reported that her condition worsened in the last week.

**Figure 1:**
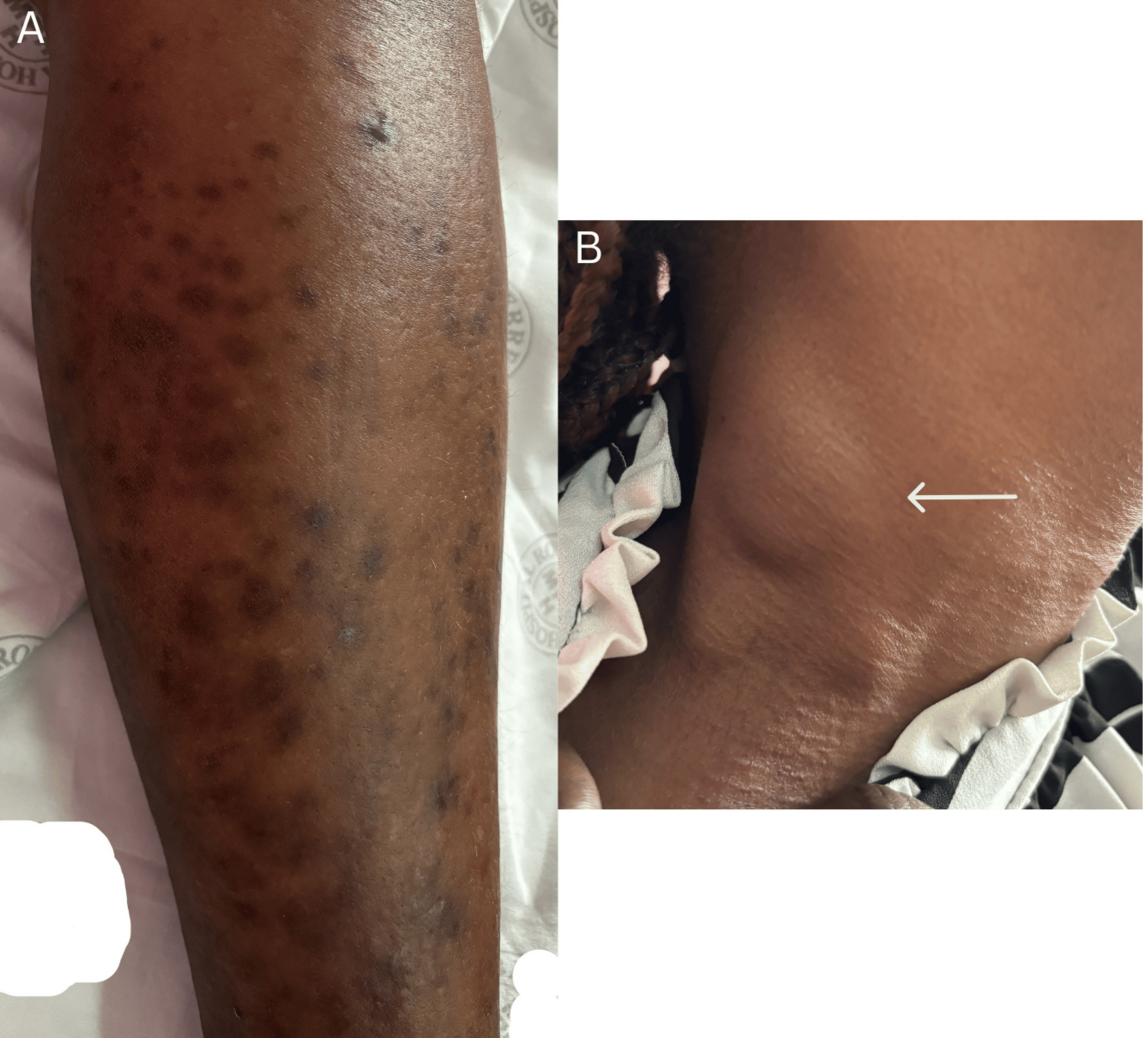
General examination findings A: Violaceous plaques and papules on patient’s left lower limb; B: Lymphadenopathy in right posterior triangle of neck (white arrow)

Physical examination revealed the following: blood pressure 99/61 mmHg, pulse rate 113 bpm, respiratory rate 22 bpm, pulse oximetry 96% on room air, and temperature of 36.7 °C. Furthermore, she had pallor of the conjunctivae; generalized lymphadenopathy involving the posterior (Figure 1), and anterior cervical, axillary, as well as the inguinal chains. The largest node was in the right axillae measuring approximately 4×3×1.5 cm. On chest examination, pleural effusion was suspected as there was dullness to percussion at the left costophrenic angle with absent breath sounds in the same area. Hepatomegaly with a liver span of 19 cm and a splenomegaly extending three cm below the costal margin was palpated. Additionally, abdominal exam revealed massive ascites with shifting dullness and a fluid thrill. Examination of the left lower limb demonstrated multiple violaceous plaques and papules (Figure 1). Moreover, there was bilateral lower limb pitting edema and more than a 2 cm discrepancy in the leg circumference of the left leg compared to the right leg. The left leg measured 40 cm at the calf and the right leg measured 36 cm; consequently, clinical suspicion for deep vein thrombosis (DVT) was raised. 

**Figure 2:**
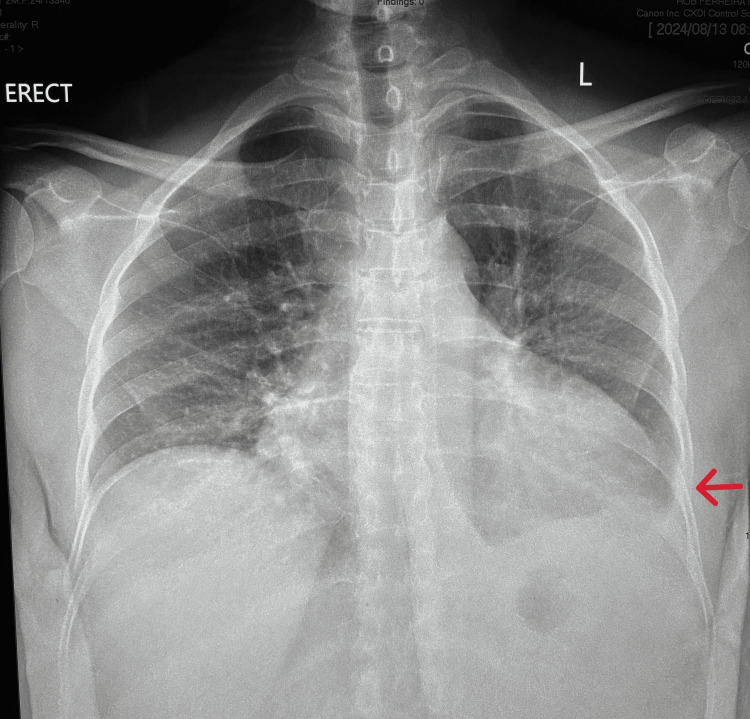
Anteroposterior chest roentgenogram demonstrating pleural effusion in left hemithorax (red arrow)

**Figure 3:**
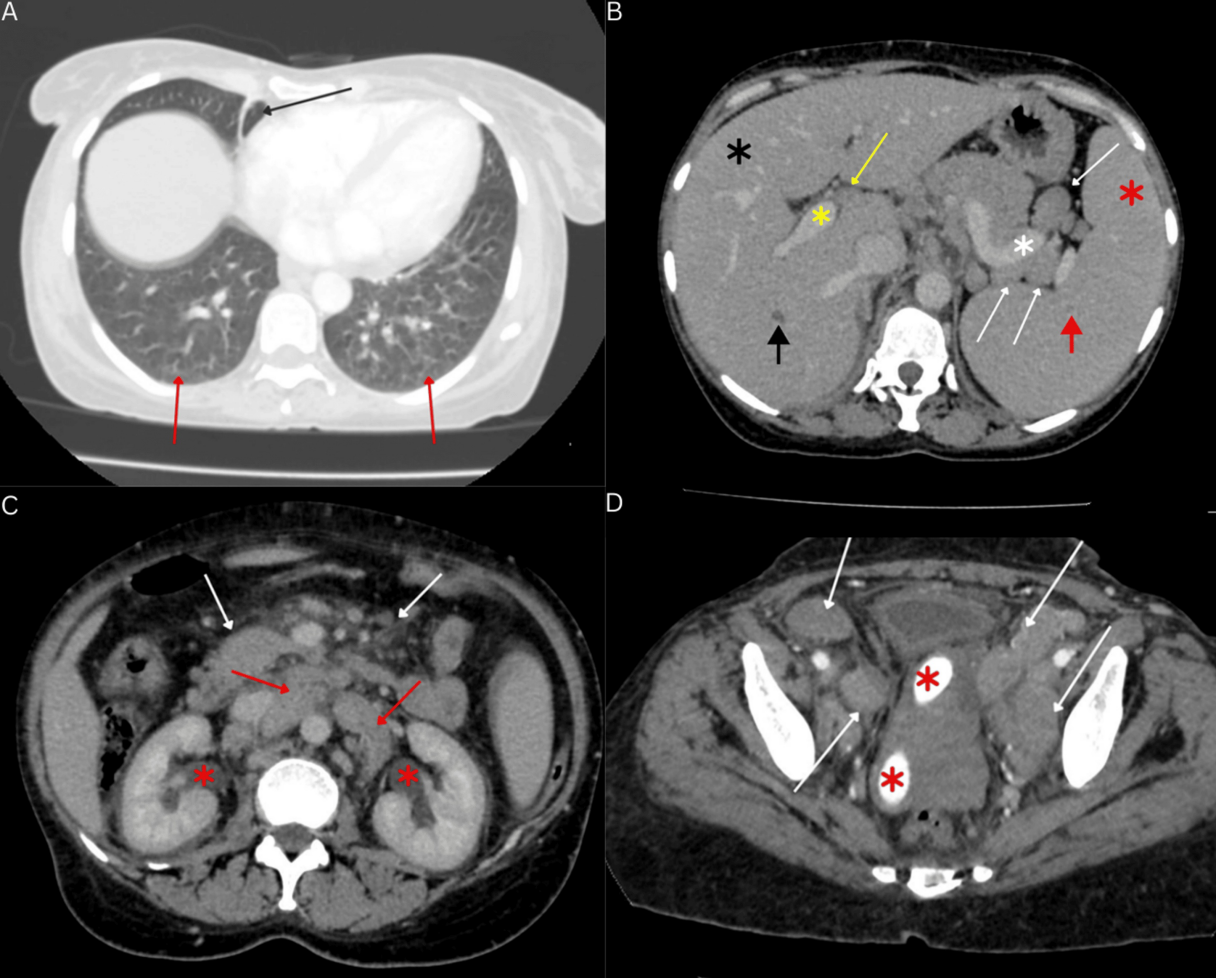
Contrast enhanced axial CT images A: CT chest with lung window demonstrates right lower lobe fibrotic band (black arrow) and diffuse smooth interlobular septal thickening (red arrows). B: Abdominal CT demonstrates hepatomegaly (black asterisk), hepatic nodule (black arrow), porta hepatis lymphadenopathy (yellow arrow), dilated portal vein (yellow asterisk), splenomegaly (red asterisk), splenic nodule (red arrow), splenic hilar lymphadenopathy (white arrow) and splenic vein dilatation (white asterisk). C: Abdominal CT demonstrates bilateral hydronephrosis (red asterisk), paraaortic (red arrows) and mesenteric lymphadenopathy (white arrows). D: Pelvic CT demonstrates bilateral internal and external iliac lymphadenopathy (white arrows) as well as an incidental uterine pessary (red asterisk) CT: computed tomography

Radiological examination demonstrated a DVT in the common femoral vein on venous Doppler of the left lower limb. Chest roentgenogram was significant for a pleural effusion in the left hemithorax (Figure 2). Echocardiogram showed normal left ventricular systolic and diastolic function with normal valvular structure and function. Computed tomography revealed a significant lymphadenopathy burden involving the cervical, thoracic, abdominal, and pelvic regions, with the largest lymph node measuring 6×5×4 cm (Figure 3). Pulmonary findings included bilateral, diffuse, smooth interlobular septal thickening, lower lobe fibrotic changes, and multiple pulmonary nodules. The liver demonstrated hepatomegaly with multiple hypodense nodules predominantly distributed along the periportal region and branches of the portal vein. Associated findings included dilation of the portosystemic system with periumbilical collateral formation. These findings are suggestive of non-cirrhotic portal hypertension, most likely secondary to obstruction by hepatic nodules and periportal lymphadenopathy. The spleen exhibited massive splenomegaly with multiple hypodense nodules. Differential diagnoses for these findings include disseminated KS, a lymphoproliferative disorder, and hemophagocytic lymphohistiocytosis (HLH). Additional incidental findings included cholelithiasis, bilateral mild hydronephrosis, and a uterine prolapse with an in-situ pessary (Figure 3). 

Biochemical investigation showed bi-cytopenia, with a white cell count of 7.25×109/L, hemoglobin of 8.6 g/dL, and a platelet count of 88×109/L (Table 1). Additionally, the patient had isolated elevated gamma-glutamyl transferase of 40 U/L, and serum albumin measured 19 g/L. C-reactive protein was 150 mg/L and erythrocyte sedimentation rate >120 mm/hour. Furthermore, she had creatinine of 45 µmol/L, blood urea nitrogen of 1.8 mmol/L, and estimated glomerular filtration rate of 112 mL/minute. The patient also had hypertriglyceridemia with triglycerides of 2.64 mmol/L. She had an iron transfer block, with ferritin of 1309 ng/mL, iron of 2 µmol/L, transferrin of <1 g/L, and % saturation of <15 %. She had a low absolute CD4 lymphocyte count of 167 cells/µL and an uncontrolled HIV viral load of 232 copies/ml. Hepatitis B and C investigations and cryptococcal antigen test were negative. EBV serology test revealed an elevation in IgG levels. Blood cultures showed no growth. Ascitic fluid analysis showed fluid protein of 30 g/L, fluid albumin of 15 g/L, fluid lactate dehydrogenase (LDH) of 78 U/L, and serum ascites albumin gradient of 4 g/L, with no organisms observed on microscopy and no growth on culture. These findings suggested ascites with normal portal pressure possibly related to infections such as tuberculosis (TB), peritoneal malignancy, pancreatitis, or nephrotic syndrome. Our team concluded that the ascites was not related to the dilated portosystemic system, which is secondary to the compressive effect of the periportal lymphadenopathy, and continued to pursue other causes. Urine analysis showed 1+ proteinuria on dipstick test, and increased protein creatinine ratio of 0.104 g/mmol creatinine, which is severely increased but not nephrotic range. 

**Table 1:** Laboratory investigations ANA: antinuclear antibody; ALT: alanine aminotransferase; ALP: alkaline phosphatase; AST: aspartate aminotransferase; BUN: blood urea nitrogen; CrAg: cryptococcal antigen; CRP: C-reactive protein; CD4: cluster of differentiation 4; DNA: deoxyribonucleic acid; EBV: Epstein-Barr virus; eGFR: estimated glomerular filtration rate; ENA: extractable nuclear antigen; ESR: erythrocyte sedimentation rate; GGT: gamma-glutamyl transferase; LDH: lactate dehydrogenase; TB NAAT: tuberculosis nucleic acid amplification test; SAAG: serum ascites albumin gradient

Analyte	Result	Reference values
White cell count	7.25×10^9^/L	3.90-12.60
Hemoglobin	8.6 g/dL	11.6-16.4
Platelet count	88×10^9^/L	186-454
BUN	1.8 mmol/L	2.1-7.1
Creatinine	45 µmol/L	49-90
eGFR	112 mL/min	>60
Albumin	19 g/L	35-45
ALT	15 U/L	5-20
AST	17 U/L	0-30
GGT	40 U/L	4-24
ALP	116 U/L	47-119
Ferritin	1309 ng/mL	30-400
Transferrin	<1 g/L	2.2-4
Iron	2 µmol/L	10-30
Transferrin saturation %	<15 %	20-50
Reticulocyte production index	2.7	
ESR	>120 mm/hr	<20
CRP	150 mg/L	<10
Triglycerides	2.64 mmol/L	<1.7
CD4 count	167 cells/µL	500-1,500
HIV viral load	232 copies/mL	<20
CrAg	Negative	
EBV IgG	35 U/ml	<18
Blood cultures	No growth	
Sputum TB NAAT	Negative	
Ascitic fluid protein	30 g/L	
Ascitic fluid albumin	15 g/L	
Ascitic fluid LDH	78 U/l	
SAAG	4 g/L	
Urine protein/creatinine ratio	0.104 g/mmol	<0.015
ANA	Negative	
Anti-double-stranded-DNA antibodies	Negative	
ENA antibodies	Negative	

A punch biopsy of the skin was done and demonstrated vaso-formative spindle cells with HHV-8 positivity of the cells. A histological diagnosis of KS in the plaque stage was made. An excisional lymph node biopsy of one of the left posterior chain nodes was performed as well. This demonstrated aggregates of large atypical lymphoid cells with vesicular chromatin and prominent nucleoli and atypical forms of mitosis. Immunohistochemistry was significant for CD3 membranous positivity of the T cells, CD138 membrane positivity, CD 20 nuclear positivity, HHV-8 nuclear positivity, MUM-1 and EBER-ISH positivity, and a Ki67 index of > 40% (Table 2). Histomorphology and immunophenotype were consistent with EPEL. Bone marrow aspirate and trephine biopsy did not show any infiltration of the marrow by a lymphoproliferative disorder or the presence of hemophagocytosis. 

**Table 2:** Immunohistochemistry of excised lymph node CD: cluster of differentiation; Ki67: antigen Kiel 67; HHV8: human herpes virus 8; ALK-1: anaplastic lymphoma kinase 1; BCL6: B cell lymphoma 6; MUM-1: multiple myeloma 1; EBER-ISH: Epstein-Barr encoding region- in situ hybridization

Analyte	Result
CD3	Membranous positivity in reactive T-cells
CD20	Nuclear positivity
Ki67	Proliferative index of greater than 40%
HHV8	Nuclear positivity
ALK-1	Negative
CD138	Membranous positivity
CD15	Negative
CD30	Negative
CD10	Negative
BCL6	Negative
MUM-1	Nuclear positivity
EBER-ISH	Nuclear positivity

Differential diagnoses 

**Figure 4:**
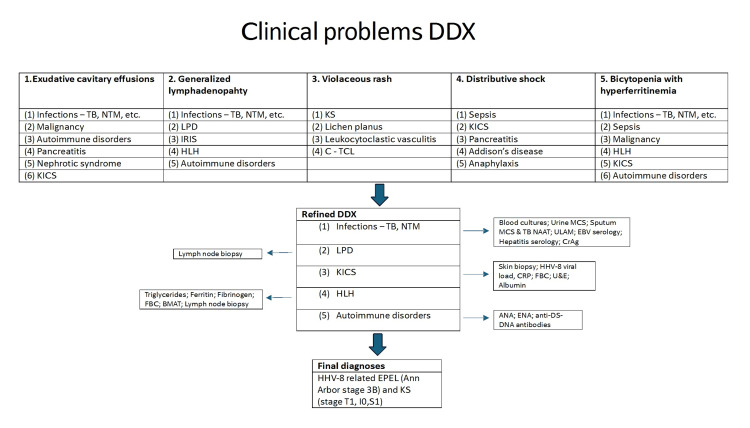
Diagnostic pathway ANA: antinucleur antibodies; BMAT: bone marrow aspirate and trephine biopsy; CRP: C reactive protein; CrAg: cryptococcal antigen; DDX: differential diagnoses; DNA: deoxyribonucleic acid; DS: double stranded; EBV: Epstein-Barr virus; EPEL: extra cavitary primary effusion lymphoma; ENA: extractable nucleur antigen; FBC: full blood count; HHV-8: human herpesvirus-8; HLH: hemophagocytic lymphohystiocytosis; IRIS: immune reconstitution inflammatory syndrome; LPD: lymphoproliferative disorders; KS: Kapsi sarcoma; KICS: kaposi sarcoma inflammatory cytokine syndrome; NTM: nontuberculous mycobacteria; TB: tuberculosis; U&E: urea and electrolytes; ULAM: urine lipoarabinomannan

Our leading differential diagnoses for the patient, at the time of admission, included (i) infectious etiologies such as extrapulmonary TB, (ii) a lymphoproliferative disorder, (iii) KS inflammatory cytokine syndrome (KICS), (iv) hemophagocytic lymphohistiocytosis (HLH), and (5) autoimmune etiologies. The differential diagnoses are very broad in an immunocompromised patient, with opportunistic infections being the most common in our setting. Our team created large differential diagnoses for each of the patient's major clinical problems and thereafter refined the differential diagnoses to the aforementioned five possibilities as shown in Figure 4.

TB is endemic in South Africa and seemed a likely culprit; however, infectious etiologies were abandoned. Laboratory investigations failed to show any evidence of infections, except for evidence of EBV infection as well as HHV-8 on histological analysis of the skin and lymph node biopsies. Our facility does not permit EBV and HHV-8 viral load testing. The patient fulfilled most criteria for KICS (Table 3) except for the presence of an elevated HHV-8 viral load. However, KICS remains a diagnosis of exclusion and is challenging to make, as a significant overlap exists in the symptomatology of KICS, HLH, and PEL. HLH was proposed as a diagnosis to explain the patient’s symptomatology. The patient fulfilled four out of eight criteria of the HLH-2004 diagnostic criteria [9]. However, bone marrow aspirate and biopsy, as well as lymph node biopsy, failed to yield evidence of hemophagocytosis; therefore, alternative diagnoses were sought. The presence of generalized lymphadenopathy, splenomegaly, cavitary effusions, and B symptoms raised clinical suspicion for a lymphoproliferative disorder. Diagnoses such as HHV-8-associated PEL, diffuse large B cell lymphoma, plasmablastic lymphoma, and Burkitt’s lymphoma were considered. Autoimmune conditions were abandoned once the results of the histological investigations were available.

**Table 3:** Working case definition of KSHV-inflammatory cytokine syndrome The working case definition of KSHV-inflammatory cytokine syndrome requires the presence of at least two clinical manifestations drawn from at least two categories (1a, b, and c), together with each of the criteria in 2, 3, and 4 KSHV: Kaposi sarcoma-associated herpesvirus; MCD: multicentric Castleman’s disease Source:  Polizzotto et al., 2012 [10]; distributed under the terms of the Creative Commons Attribution License CC BY-NC 3.0 Attribution-Noncommercial 3.0 Unported

1. Clinical Manifestations
a. Symptoms: fever, fatigue, cachexia, edema, respiratory symptoms, gastrointestinal disturbance, myalgia and arthralgia, altered mental state, neuropathy with or without pain
b. Laboratory abnormalities: anemia, thrombocytopenia, hypoalbuminemia, hyponatremia
c. Radiographic abnormalities: lymphadenopathy, splenomegaly, hepatomegaly, body cavitary effusions
2. Evidence of systemic inflammation
Elevated C-reactive protein (≥3 g/dL)
3. Evidence of HHV-8 viral activity
Elevated KSHV viral load in plasma (≥1000 copies/mL) or peripheral blood mononuclear cells (≥100 copies/10^6^ cells)
4. No evidence of HHV-8 associated Castleman’s disease
Exclusion of MCD requires histopathologic assessment of lymphadenopathy if present

EPEL shows a higher frequency of expression of B-cell markers, such as CD20, as well as an increased frequency of aberrant expression of T-cell markers like CD3 [7], as was the case with our patient. However, the diagnosis of PEL was confirmed with immunohistochemical staining for latency-associated nuclear antigen 1. The former is an HHV-8-associated latent protein positive in the nuclei of PEL cells [7], as was the case in our patient. Additionally, HIV/AIDS-associated PEL is characterized by a distinct profile of combined features of EBV-transformed lymphoblastoid cell lines and plasma cells. Our patient expressed CD138 and MUM-1 positivity, which are plasma-cell-associated markers, as well as EBER-ISH positivity in keeping with HIV/AIDS-related PEL [7]. Our team decided on the final diagnoses of HHV-8-related EPEL (Ann Arbor stage 3B) and KS (stage T1, I0, S1) in a person with AHD. 

Treatment 

The patient initially received empiric antibiotic treatment with intravenous ceftriaxone, later escalated to meropenem for presumed distributive shock due to sepsis. This was later discontinued due to the absence of any infection from the results of the laboratory investigations. The patient's condition necessitated intermittent resuscitation in the form of intravenous fluids and blood products. Once the final diagnosis of EPEL (Ann Arbor stage 3B) and KS (stage T1, I0, S1) was made, the patient was referred and transferred to the oncology department for definitive management of her condition. It was decided to initiate the patient with a three-weekly cycle of rituximab, cyclophosphamide, doxorubicin, vincristine, and prednisolone (R-CHOP) regimen. A treatment course of six cycles was prescribed for the patient. Additionally, prophylaxis for *Pneumocystis jirovecii* with bactrim and ART was continued. The bactrim prophylaxis will be continued until the patient's CD4 count is above 200 cells/µL and throughout the patient's chemotherapy regimen. This is according to national guidelines and the hospital's practice guidelines. The patient’s viral load at the time of R-CHOP initiation was lower than the detectable limit. 

## Discussion

The intersection of KS and EPEL in a single patient with HIV presents a unique clinical challenge. Both conditions are associated with advanced immunosuppression and are linked to HHV-8. As evidenced by this case, a significant diagnostic challenge exists in delineating between pathologies. This discussion explores the implications, management strategies, and outcomes of patients who present with both malignancies. 

The co-occurrence of KS and EPEL in PLHA is rare but highlights the complex interplay of viral oncogenesis and immunosuppression. In PLHA, HHV-8 can lead to the transformation of infected cells into malignant counterparts [2]. The immunosuppressive effects of HIV facilitate the replication and pathological progression of HHV-8, increasing the risk for both KS and PEL. The presence of HIV not only impairs the immune response but also creates an environment conducive to the development of these malignancies. 

Patients presenting with both KS and PEL may exhibit a range of symptoms, including visible skin lesions from KS and respiratory distress or abdominal discomfort due to fluid accumulation from PEL [11]. This dual presentation complicates diagnosis and management, as the symptoms of one malignancy may mask or mimic that of the other. A high index of suspicion is required for both conditions, particularly in immunocompromised patients [4]. KS-immune reconstitution syndrome (IRIS) should also be considered in patients starting ART recently, within three months, as this condition can present in a similar manner. Volkow et al. found the median time of diagnosis of KS-IRIS to be 10 weeks after ART initiation [12]. This was not part of our patient's differential diagnoses, as our patient had been on ART for the last four years.

The management of concurrent KS and PEL in PLHA requires a multidisciplinary approach. Initiating or optimizing ART is crucial for improving immune function and potentially reducing the progression of both malignancies [11,13]. Glucocorticoids are not part of the treatment regimen in KS. The use of glucocorticoids is a risk factor for KS-IRIS and significantly increases KS-related mortality [14]. Treatment options for KS may include localized therapies such as radiation, systemic chemotherapy, or immunotherapy, depending on the extent and severity of the disease. PEL typically requires systemic chemotherapy, often using regimens such as CHOP, although treatment outcomes can be poor given the aggressive nature of this lymphoma [15]. Rituximab was included in our patient’s treatment regimen due to the CD20 positivity of the tumor. Symptomatic management for complications arising from either malignancy, such as pain control and management of effusions, is essential. 

The prognosis for patients with simultaneous KS and PEL is poor, due to the advanced stage of disease at diagnosis and underlying immunosuppression [16]. However, aggressive management, including ART and tailored oncological therapy, can lead to improved outcomes.

According to Hickam's dictum, a patient can present with multiple different diagnoses simultaneously. Prudent application of diagnostic parsimony, also known as Occam’s razor, may suffice in the majority of cases. However, the complexity of our clinical case demonstrates that Hickam’s dictum should be applied when such diagnostic dilemmas exist [17].

## Conclusions

In AHD, it is appropriate to have a large differential of possibilities to explain their symptoms. Hickam’s dictum, rather than Occam's razor, should be the preferred diagnostic approach for these patients. Although infections are common and important etiological considerations, clinicians should maintain a high index of suspicion for malignancy, autoimmune, and autoinflammatory conditions in PLHA. As the landscape of HIV treatment continues to evolve, ongoing research into the best practices for managing multiple malignancies in this particular population is essential. 
